# Applying Eye Movement Modeling Examples to Guide Novices’ Attention in the Comprehension of Process Models

**DOI:** 10.3390/brainsci11010072

**Published:** 2021-01-07

**Authors:** Michael Winter, Rüdiger Pryss, Thomas Probst, Manfred Reichert

**Affiliations:** 1 Institute of Databases and Information Systems, Ulm University, 89081 Ulm, Germany; manfred.reichert@uni-ulm.de; 2 Institute of Clinical Epidemiology and Biometry, University of Würzburg, 97070 Würzburg, Germany; ruediger.pryss@uni-wuerzburg.de; 3 Department for Psychotherapy and Biopsychological Health, Danube University Krems, 3500 Krems, Austria; thomas.probst@donau-uni.ac.at

**Keywords:** Business Process Models, Process Model Comprehension, Eye Movement Modeling Examples, eye tracking, human-centered design, cognition

## Abstract

Process models are crucial artifacts in many domains, and hence, their proper comprehension is of importance. Process models mediate a plethora of aspects that are needed to be comprehended correctly. Novices especially face difficulties in the comprehension of process models, since the correct comprehension of such models requires process modeling expertise and visual observation capabilities to interpret these models correctly. Research from other domains demonstrated that the visual observation capabilities of experts can be conveyed to novices. In order to evaluate the latter in the context of process model comprehension, this paper presents the results from ongoing research, in which gaze data from experts are used as Eye Movement Modeling Examples (EMMEs) to convey visual observation capabilities to novices. Compared to prior results, the application of EMMEs improves process model comprehension significantly for novices. Novices achieved in some cases similar performances in process model comprehension to experts. The study’s insights highlight the positive effect of EMMEs on fostering the comprehension of process models.

## 1. Introduction

Flow charts are visual representations delineating algorithms, systems, or processes [1,2,3]. They are widely used in different domains (e.g., healthcare, computer science, and business) for the following purposes: documentation, instant communication, effective analyses, and problem solving [2,4,5]. Process models constitute a derivation of flow charts for the graphical documentation of processes [6,7]. A process model represents all activities to achieve a specific objective. For example, an order to cash (O2C) process describes all activities for receiving and processing customer orders for goods and services [8]. An activity consumes resources (e.g., machines) to convert inputs (e.g., data) into outputs (e.g., value). In more detail, a process model depicts all activities, decisions, and involved stakeholders as well as resources in a process [9]. In order to make use of the merits of process models, the understanding of such models (i.e., process model comprehension) should not pose any difficulties for the involved stakeholders [10]. Many unresolved issues concerning the factors thwarting the comprehension of process models exist, and therefore the identification of these factors is decisive. For this reason, a prerequisite for an overall comprehension of processes is to ensure that all stakeholders can easily read and comprehend corresponding process models in an efficient and effective manner [11].

Despite existing research in the field of process model comprehension, stakeholders, both inexperienced and experienced, are still facing challenges on how to properly read and comprehend process models [12]. Numerous works in the literature exist with respect to process model comprehension. For example, [13] presents results from a series of experiments about factors having an impact on process model comprehension. An extensive literature overview about empirical work focusing on process model comprehension is consolidated in [14], in which objective and subjective factors influencing process model comprehension are discussed.

In this context, to foster process model comprehension, the usage of eye tracking has proven to be a suitable methodology that may yield promising insights [15]. Process model comprehension strategies can be visualized (e.g., depiction of the path followed by the eyes when reading a model) and difficult to comprehend modeling constructs (e.g., loops) can be identified in a process model [16]. The latter is identified by analyzing modeling constructs to which the eyes repeatedly jump back frequently, or to which the eyes have a longer average dwell time. Different types of eye movements (i.e., fixations and saccades) may serve as an indicator representing emerging cognitive load during process model comprehension (e.g., more fixations during high load) [17]. In addition, the authors of [18] discussed experiences and lessons learned from eye tracking studies on process model comprehension. The approach discussed in [19] explains how model-related (e.g., size of a process model) and person-related (i.e., process modeling experience) factors of process model comprehension are influenced by visual cognition variables (e.g., scan path). The results shown in [20] indicate that visual cues (e.g., colors) used in process models improve the overall comprehension. Finally, the authors of [21] investigated the impact of coloring in decision models.

**Figure 1:**
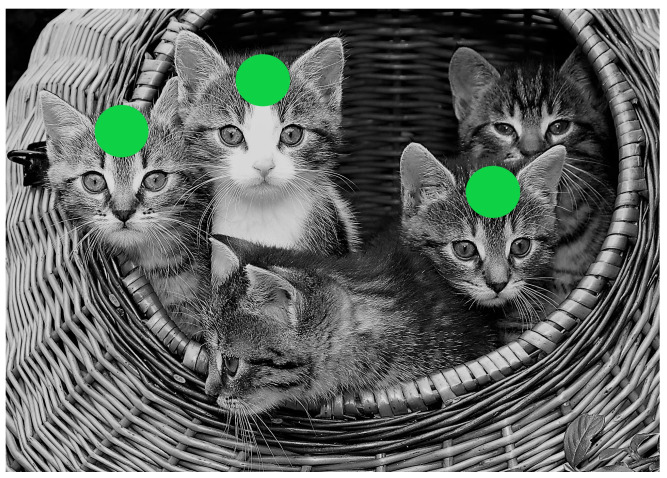
Eye Movement Modeling Examples (EMME) in dot display condition.

If factors that hamper proper process model comprehension are not properly addressed, the respective processes might not deliver the required outcomes. Failures that happen in the application of such models have been commonly linked to model incomprehension [22]. As a consequence, the identification of factors, both positive and negative, that influence the comprehension of such models is essential. For the continuation of ongoing research on process model comprehension, this paper presents the results obtained from a second study of the authors (i.e., Study Two), which is part of a three-stage study to foster the comprehension of process models (see Section 5). In the first study (i.e., Study One), we analyzed eye movements and visualized comprehension strategies of novices and experts while reading and comprehending process models. The results revealed that there were similarities and differences between experts and novices in process model comprehension. For example, all participants tried to find the starting point in the process model when they first gazed at the model [18]. It was particularly noticeable that experts did not look at all the individual elements in the shown process models juxtaposed with novices. The results further showed that experts comprehended process models more efficiently than novices [23]. Based on these findings, the question emerged whether visual observation capabilities of experts during the comprehension of process models in Study One can be efficiently conveyed to novices. Based on the eye tracking data obtained from the experts in Study One, we created Eye Movement Modeling Examples (EMMEs) for a second study (i.e., topic of this paper), which shall assist novices in the comprehension of process models [23]. EMMEs are instruments to teach and improve performance on perceptual tasks [24]. The basic idea behind an EMME is to convey mandatory visual observation capabilities for perceptual tasks, especially to novices [25]. Therefore, eye movements of experts were recorded during a perceptual task and, afterwards, their eye movements (e.g., fixations) were displayed during novices’ performance of a task [26]. EMMEs are usually superimposed in a dynamic multimedia form (e.g., video) [27]. However, it is also possible to superimpose the eye movements (e.g., fixations) of experts in static pictures. These eye movements could, on the one hand, appear for a short period of time in a picture to attract the gaze of the viewer or, on the other hand, be displayed permanently to guide the viewers’ gaze. For example, Figure 1 presents an EMME, which depicts an image of cats with three superimposed dots (i.e., dot display condition).

Crucial parts in the image are highlighted with green dots to visually attract the attention of the viewer. Research showed that this kind of teaching method with EMMEs can improve performance on perceptual tasks and fosters learning [28]. The research presented in [29] reveals a positive effect of EMMEs on comprehension strategies in medical image diagnosis. The authors in [30] are using EMMEs for the improvement of information processing and learning from pictures and texts. [27] provides evidence that EMMEs change the information processing during learning and fosters the performance in learning especially for learners with lower skills. The work presented in [31] evaluated the application of EMMEs during computer programming, resulting in an improved solving of programming problems. Finally, in [32], the authors demonstrated that EMMEs can be effectively used in order to guide reading and comprehension strategies. In [33], the same authors presented how EMMEs raise attention during the critical reading of web pages.

To conclude, the following three research questions (RQ) are addressed in study presented in this paper (i.e., Study Two of the three-stage study described earlier):

- **RQ 1**: Do novices perform better in process model comprehension when the novices are supported by EMMEs, and does this depend on the complexity of the process model?
- **RQ 2**: Do novices perform differently as experts in process model comprehension when the novices are supported by EMMEs, and does this depend on the complexity of the process model?
- **RQ 3**: Do novices perform differently in process model comprehension when they are supported by different conditions of EMMEs, and does this depend on the complexity of the process model?

In RQ 1, the results obtained from novices of Study One were juxtaposed with the results of novices from the current study [23]. We wanted to investigate with RQ 1 whether the application of EMMEs is beneficial to foster the comprehension of process models.

In RQ 2, the results from novices of the current study were compared with results from experts of Study One [23]. Therefore, RQ 2 is concerned with the question whether novices supported by EMMEs performed differently (e.g., similar) in process model comprehension juxtaposed with experts.

Finally, inRQ 3, the results of novices being confronted with different conditions of EMMEs were compared to each other. An EMME can reflect different conditions (see Figure 1), depending on the focus being set. We wanted to reveal with RQ 3 whether different conditions of EMMEs may pose varying effects on process model comprehension.

In all three RQs, we took the level of complexity of the process models into account. Therefore, the participants worked with easy, medium, and hard process models.

To the best of our knowledge, there exist no other works dealing with the application of EMMEs in the context of process model comprehension so far.

The structure of this paper is as follows: Section 2 provides information about materials and methods of the conducted study. In Section 3, obtained results of the study are presented descriptively, including significance tests. The analyzed results are discussed in Section 4, including implications for practice and research as well as limitations. Finally, Section 5 summarizes the paper and discusses future work.

## 2. Materials and Methods

### 2.1. Participants

The study at hand included 43 participants in total. Eighteen were female and the mean age was 22.69 years (SD=2.13). A prerequisite for the study was that participants had to have little or no experience in process modeling for the successful application of EMMEs. By using a specific 5-point Likert scale from Study One, participants were asked for prior experience in process modeling (i.e., ranging from not at all experienced (0) to highly experienced (4)); to ensure that the prerequisite for the participation in the study was met. Novices who have already participated in Study One were not allowed to participate in this study. All participants were recruited at Ulm University and were composed of research assistants and students from different disciplines like computer science and economics. Table 1 summarizes the sample description and comparison in baseline variables. The table shows the baseline variables for novices of this study, which were split with the round-robin approach (i.e., alternating assignment to one of the two groups in order to ensure a balanced distribution) into two groups, i.e., dot (Sample Dot) and path (Sample Path) display condition. The prior obtained results of novices (Sample Novice) and experts (Sample Expert) of Study One (i.e., gray background) are depicted in Table 1 as well [23]. Students with high prior knowledge in process modeling and professionals having practical experience in working with process models were referred as experts in the Sample Expert. More specifically, the experts from respective sample were familiar with the modeling notation Business Process Model and Notation (BPMN) 2.0 [34]. On the one hand, they were confident in comprehending process models created with the BPMN 2.0, and on the other hand, they felt competent in applying BPMN 2.0 for the modeling of processes. Finally, the experts have already spent at least 20 hours on process modeling and comprehending.

### 2.2. Materials

**Figure 2:**
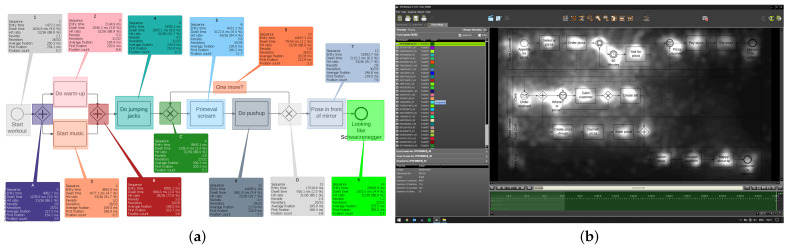
Identification and creation of EMMEs. (**a**) Areas of interest (AOIs) with KPIs; (**b**) Focus map.

For the current study, the same process models as in Study One were used. These were three process models expressed in terms of the BPMN 2.0, divided up into three levels of complexity (i.e., easy, medium, and hard): The easy process model was only composed of basic modeling elements. With rising level of complexity, new BPMN elements were added and the total number of model elements was increased as well. Regarding the process scenario, the easy process model described a workout process (i.e., sequential process). An auction process is documented in the process model reflecting a medium level of complexity (i.e., a process involving a communication with two participants). Finally, the hard process model shows the process of a pizza order (i.e., a process with numerous decisions and participants). Based on the results obtained from Study One, we enriched the process models with eye movements derived from experts, whence the EMMEs were created. In more detail, eye movement parameters (i.e., fixations and scan paths) obtained from experts in Study One were superimposed on respective process models. Two EMME versions reflecting different conditions were created, i.e., dot and path display condition. The dot display condition mainly refers to the syntactical dimension (i.e., compliance with process modeling rules) of a process model. Therefore, specific modeling parts were highlighted with colored dots in the EMMEs, which were relevant for a proper process model comprehension. For example, the colored dot should target the eyes on modeling elements indicating a decision, in which the process flow was split and only those activities were executed that were in the flow of the positive decision, whereas the other activities could no longer be executed. The placement of the colored dots in the models was determined during the analysis of the most frequent fixations obtained in Study One from experts. More specifically, as demonstrated by the authors in [16], experts consider only relevant parts (e.g., activities or modeling structures) in a process model during comprehension (e.g., in answering questions). These relevant parts, which would have to be considered for the correct answering of comprehension questions, were identified descriptively and visually by the definition of specific areas of interest (AOIs) using SMI BeGaze software. An AOI is a method to select regions within a presented stimulus (e.g., image) in order to extract key performance indicators (KPIs such as fixations, saccades) specifically for those regions [35]. Separate AOIs were defined for all individual modeling elements in the three process models in order to identify the most frequent fixations of experts in Study One, which were relevant for the proper comprehension of respective models. The path display condition mainly focuses on the semantic dimension (i.e., correct and complete documentation of the process scenario) of a process model. This should ensure that all semantically relevant information (e.g., activities) were considered. For the illustration of the path display condition, experts course of their eyes (i.e., scan path) obtained in Study One were shown on the process model in respective EMMEs. The scan path represents the average chronological concatenation of experts’ eye movements during the comprehension of process models. The identification of the average scan path was done within BeGaze software, in which the sequence of consideration of the defined AOIs has been reproduced. In addition to the KPIs, the experts’ eye movements were visually analyzed with specific visualization techniques such as heat and focus maps [36]. Those techniques do not present information about single eye movement events (e.g., fixation), but reveal the focus of visual attention in a stimulus for all participants at a time. In general, both conditions ensured that a process model could be comprehended properly in order to be able to answer the comprehension questions. Figure 2 present exemplary excerpts from BeGaze Software showing AOIs with respective KPIs (see Figure 2a) and a focus map (see Figure 2b) on used process models.

**Figure 3:**
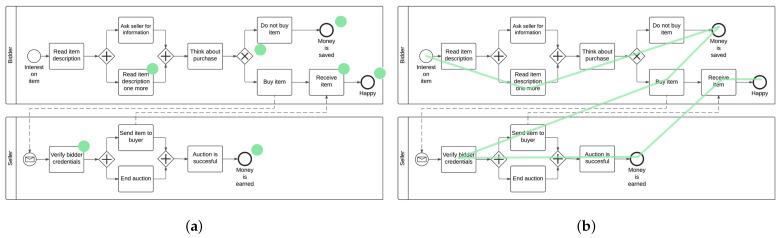
Eye Movement Modeling Examples (EMMEs). (**a**) Dot display condition; (**b**) Path display condition.

Note that the two EMME conditions (i.e., dot and path) were static and were permanently shown on the process models. The intention was to provide a visual guidance for the reader during the comprehension of respective process models. In the beginning, we put an emphasis on the evaluation of the dot and path display condition. These two conditions were particularly well suited in our context, because the focus can be separately set on the syntactic and semantic dimension in a process model. Figure 3 presents the two EMME conditions on the medium process model used in the study, i.e., dot (see Figure 3a) and path display condition (see Figure 3b). The same four *true-or-false* comprehension questions for each process model referring on model semantics and syntactics from Study One were used. These comprehension questions were used to evaluate whether the process models were comprehended properly by novices in order to investigate the effects of EMMEs (see Appendix A).

### 2.3. Instrumentation

Demographic data (e.g., age, gender, process modeling experience) was collected with paper-based questionnaires. For capturing eye movements, eye tracking was performed with the SMI iView X Hi-Speed system. Equal to Study One, the tracking device was placed in front of a 23” monitor (resolution of 1920 × 1080, 96 PPI) showing the EMMEs to the participants. For calibration, a 13-point calibration was performed. Eye movements were recorded at a sampling rate of 240 Hz. Participants used a keyboard with two predefined keys providing the respective answering options (i.e., “true” and “false”) for the answering the comprehension questions. Eye tracking data collected during the study was analyzed and visualized with SMI BeGaze 3.7.59 software. Finally, SPSS 25 was used for all statistical analyses.

### 2.4. Performance Measures

The following performance measures were considered in Study Two:

Fixation: Fixations constitute eye movements of very low velocity at a specific point in a stimulus (e.g., picture), in which relevant information is extracted about what is being looked at [37]. The measuring of the number of fixations allows us to make conclusion about specific points (e.g., process modeling constructs) in the stimulus (i.e., process model) that may pose a challenge in the comprehension process for the participants (e.g., due to frequently recurring fixations).

Fixation duration: The fixation duration indicates the period of time, in which the eyes remain relatively still while looking at a stimulus [38]. During this period of time, the acquisition of information from the currently viewed point in a stimulus takes place. The analysis of the average fixation duration allows for assumption regarding the mental load (e.g., higher duration at higher load) during process model comprehension [39].

Scan path: The scan path is composed of the concatenation of eye movements (i.e., fixations and saccades) in order to reflect the path the eyes take while analyzing a stimulus [40]. The analysis of the scan paths reveal distinct eye movement patterns (e.g., back-and-forth saccade jumps) of the participants.

Score: Participants needed to answer for each comprehended process model four true-or-false comprehension questions to check the effectiveness of the EMMEs. The comprehension questions referred to the semantic and syntactic dimension of the process model. A point was awarded for each correct given answer, i.e., a participant could score a maximum of four points per process model.

Duration: A timestamp was added at the moment participants started comprehending respective process models. After comprehending a process model and answering respective comprehension questions, another timestamp was added. This allowed us to measure the duration needed on a fine-grained level.

### 2.5. Study Design

**Figure 4:**
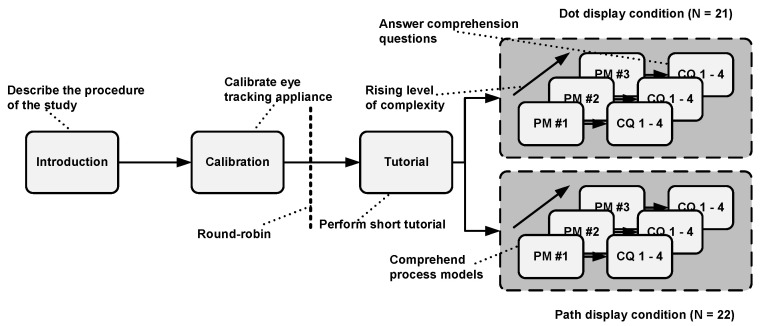
Design used in this study.

The study design is based on the guidelines set out by [41]. The study was conducted in a designated lab at Ulm University. Prior to the actual study, a pilot study with six participants was conducted to review whether our study design and the study material were appropriate for the study. Only one participant could be evaluated at each study session and a session took about 30 min. Equal to Study One, a study session was as follows: The participant was welcomed and the study procedure (i.e., purpose and objective) was explained. In more detail, the participant was advised to pay attention for visual cues (i.e., dot or path display condition) in the presented process models and to perform the study task (i.e., process model comprehension and answering of comprehension questions) as quickly, but at the same time, as carefully as possible. These instructions were given in order to mitigate potential risks, which could have distorted the results (e.g., achieving better results through longer time taking). The participant was asked afterwards to answer a demographic questionnaire for the purpose of collecting relevant demographic data (e.g., age, gender, process modeling experience) and to check whether the prerequisite (i.e., having no or less experience in working with process models) for participation in the study was guaranteed. Following this, the eye tracker was calibrated with a 13-point calibration to ensure a precise recording of the eye movements. After completing all mandatory steps, an EMME condition (i.e., dot or path display condition) was selected using the round-robin approach (i.e., alternating assignment to the dot and path display condition) in order to ensure a balanced distribution of both conditions across all participants. Consequently, a participant saw either the dot or path display condition in all the EMMEs. Then, the participant completed a brief tutorial in order to familiarize themselves with the usage of the eye tracker and the procedure of the study. A simple process model without any visual cues was shown and the participant needed to answer two comprehension questions regarding the shown model using a keyboard with two predefined keys reflecting the answering options “true” and “false” in the tutorial. After completing the tutorial, the participants needed to comprehend the single EMMEs (i.e., dot or path display condition). The respective EMME condition was displayed on the process model during the entire task. First, the EMME with the easy process model was shown, followed by the medium and the hard process model. After each evaluated process model, the participant had to answer four true-or-false comprehension questions with the keyboard related to the respective process model. The questions referred to process model syntactics and semantics and were used, on the one hand, in order to ensure that the participants studied the EMME, and on the other hand, to evaluate the performance in process model comprehension. Figure 4 presents the study design.

Finally, all participants gave their informed consent for inclusion before they participated in the study. This study was performed in line with the principles of the Declaration of Helsinki. All materials and methods were approved by the Ethics Committee of Ulm University and were carried out in accordance with the approved guidelines (#234/19).

## 3. Results

The following specified terms will represent respective samples:

- Sample Dot = dot display condition.
- Sample Path = path display condition.
- Sample Both = merged results from sample dot and path.
- Sample Novice = results from novices of study one.
- Sample Expert = results from experts of study one.

The following Table 2 presents mean (M) and standard deviation (SD) of the obtained results of Samples Dot and Path separately and Sample Both together. The table shows the results for Samples Novice and Expert that we obtained from Study One as benchmark [23]. The table presents the five considered performance measures, namely, the number of fixations, the average fixation duration (in ms), and resulting scan path lengths (in px). The achieved score for respective comprehension questions (max. is 4) and the duration (in ms) needed for comprehension are listed in the table for each level of complexity, i.e., easy, medium, and hard.

### 3.1. Inferential Statistics

Analyses of variance for repeated measurements were performed for each performance measure (i.e., fixation, fixation duration, scan path, score, and duration) to evaluate whether the reported descriptive results reach statistical significance. The within-subject factor was level of complexity and was comprised of three levels (i.e., easy, medium, and hard). The between-subject factor had two levels and consisted of the sample comparison of interest in the specific research question (i.e., RQ 1: Both vs. Novice; RQ 2: Both vs. Expert; RQ 3: Dot vs. Path). The *main effects* for level of complexity (ME 1) and for the respective sample comparison (ME 2) and the complexity*sample comparison interaction effect (IE) were analyzed. Finally, *repeated contrasts* were employed in the event of significance for ME 1 as well as the interaction effect (IE). The statistical tests were performed two-tailed and the significance value was set to *p* < 0.05.

The single results between the two EMME conditions (i.e., dot (Sample Dot) and path (Sample Path) display condition) are very similar. An increase in the number of fixations as well as resulting scan path is discernible with rising level of complexity. A decrease in the answering score and a longer comprehension duration is accompanied by a rising level of complexity. Comparing results from Sample Both with the results obtained from Sample Novice, an improvement in every performance measure is noticeable. Participants from Sample Both needed less fixations and had a shorter average fixation duration during process model comprehension and resulting scan paths are reflecting shorter paths. Moreover, participants from Sample Both achieved a better answering score in average and needed less duration for the comprehension of respective process models compared to Sample Novice. Comparing results from Sample Both with the results from Sample Expert, it is recognizable that the results are closer and may converge. Occasionally, the participants from Sample Both outperform Sample Expert. Only minimal differences are noticeable in each performance measure when juxtaposing the results from Samples Dot and Path.

#### 3.1.1. Results for RQ 1

Table 3 shows the results of all performance measures (i.e., fixation, scan path, score, and duration) with respect to RQ 1.

Regarding fixation, ME 1 was significant and repeated contrasts showed that the medium process model (*p* = 0.001; $\eta_{p}^{2}=0.78$) had more fixations (M = 177.77 (36.43)) than the easy process model (M = 115.35 (26.47)) and the hard process model (*p* = 0.001; $\eta_{p}^{2}=0.60$) had more fixations (M = 229.87 (55.37)) than the medium process model. ME 2 reached significance (*p* = 0.001; $\eta_{p}^{2}=0.29$) and Sample Novice had more fixations across all levels of complexity than Sample Both.

Regarding fixation duration, ME 1 was significant and repeated contrasts showed that the medium process model (*p* = 0.001; $\eta_{p}^{2}=0.63$) had a longer average fixation duration (M = 222.99 (23.18)) than the easy process model (M = 210.05 (43.68)) and the hard process model (*p* = 0.001; $\eta_{p}^{2}=0.29$) had a longer average fixation duration (M = 235.18 (47.78)) than the medium process model. ME 2 reached significance (*p* = 0.050; $\eta_{p}^{2}=0.07$) and Sample Novice had a longer average fixation duration across all levels of complexity than Sample Both. IE was also significant and repeated contrasts showed that easy process model*sample comparison vs. medium process model*sample comparison was not significant (*p* = 0.071; $\eta_{p}^{2}=0.06$) and medium process model*sample comparison vs. hard process model*sample comparison was not significant (*p* = 0.099; $\eta_{p}^{2}=0.05$) either. To further explain this IE, *t-tests* for independent samples were performed between Sample Both and Sample Novice for each level of complexity to evaluate whether there are differences between the samples in some complexity levels but not in others. Only comparison for the hard process model (*p* = 0.016; d = 0.64) reached significance indicating that the difference between novices not supported by EMMEs and those supported depend on the complexity of the process models resulting in a significant difference of the average fixation duration between Sample Novice and Sample Both in the hard process model.

Regarding scan path, ME 1 was significant and repeated contrasts showed that the medium process model (*p* = 0.001; $\eta_{p}^{2}=0.77$) had longer scan paths (M = 41,318.83 (10,737.51)) than the easy process model (M = 23,257.98 (4326.68)), but the hard process model (*p* = 0.673; $\eta_{p}^{2}=0.00$) did not have longer scan paths (M = 42,165.43 (20,400.78)) than the medium process model. Moreover, ME 2 reached significance (*p* = 0.001; $\eta_{p}^{2}=0.19$) and Sample Novice had longer scan paths across all levels of complexity than Sample Both.

Regarding score, ME 1 was significant and repeated contrasts showed that the medium process model (*p* = 0.613; $\eta_{p}^{2}=0.00$) did not have a lower score (M = 2.66 (0.95)) than the easy process model (M = 2.73 (0.90)), but the hard process model (*p* = 0.001; $\eta_{p}^{2}=0.28$) had a lower score (M = 2.18 (1.07)) than the medium process model. ME 2 was significant (*p* = 0.001; $\eta_{p}^{2}=0.52$) and Sample Novice had a lower score across all levels of complexity than Sample Both. Further, IE was also significant and repeated contrasts showed that easy process model*sample comparison vs. medium process model*sample comparison was not significant (*p* = 0.826; $\eta_{p}^{2}=0.00$), whereas medium process model*sample comparison vs. hard process model*sample comparison was significant (*p* = 0.006; $\eta_{p}^{2}=0.13$). These results show that the difference between novices not supported by EMMEs and those supported depend on the complexity of the process models. To further explain this IE, *t-tests* for independent samples were performed between Sample Both and Sample Novice for each level of complexity to evaluate whether there are differences between the samples in some complexity levels but not in others. However, all three comparisons reached significance: for the easy process model (*p* = 0.004; d = 1.00), medium process model (*p* = 0.002; d = 1.15), and hard process model (*p* = 0.001; d = 2.55). The largest difference between Sample Novice and Sample Both was, however, in the hard process model. In particular, the score of Sample Novice decreased more between the medium process model and the hard process model than the score of Sample Both.

Regarding duration, ME 1 was significant and repeated contrasts showed that the medium process model (*p* = 0.001; $\eta_{p}^{2}=0.55$) had a longer duration (M = 52,073.40 (19,113.59)) than the easy process model (M = 33,480.43 (10,555.24)) and the hard process model (*p* = 0.003; $\eta_{p}^{2}=0.14$) had a longer duration (M = 64,560.42 (19,798.52)) than the medium process model. Additionally, ME 2 reached significance (*p* = 0.001; $\eta_{p}^{2}=0.24$) and Sample Novice had a longer duration across all levels of complexity than Sample Both.

#### 3.1.2. Results for RQ 2

Table 4 shows the results of all performance measures (i.e., fixation, scan path, score, and duration) with respect to RQ 2.

Regarding fixation, ME 1 was significant and repeated contrasts showed that the medium process model (*p* = 0.001; $\eta_{p}^{2}=0.87$) had more fixations (M = 165.98 (27.31)) than the easy process model (M = 105.48 (19.26)) and the hard process model (*p* = 0.001; $\eta_{p}^{2}=0.71$) had more fixations (M = 213.05 (31.68)) than the medium process model.

Regarding fixation duration, ME 1 was significant and repeated contrasts showed that the medium process model (*p* = 0.001; $\eta_{p}^{2}=0.66$) had a longer average fixation duration (M = 215.82 (39.00)) than the easy process model (M = 203.25 (38.03) and the hard process model (*p* = 0.003; $\eta_{p}^{2}=0.14$) had a longer average fixation duration (M = 223.74 (39.80)) than the medium process model.

Regarding scan path, ME 1 was significant and repeated contrasts showed that the medium process model (*p* = 0.001; $\eta_{p}^{2}=0.73$) had longer scan paths (M = 38,368.89 (10,059.42)) than the easy process model (M = 22,142.02 (6269.76)), but the hard process model (*p* = 0.135; $\eta_{p}^{2}=0.04$) did not have longer scan paths (M = 39,416.74 (7991.86)) than the medium process model.

Regarding score, ME 1 was significant and repeated contrasts showed that the medium process model (*p* = 0.256; $\eta_{p}^{2}=0.02$) did not have a lower score (M = 3.10 (.78)) than the easy process model (M = 3.21 (0.77)), whereas the hard process model (*p* = 0.006; $\eta_{p}^{2}=0.12$) had a lower score (M = 2.76 (0.78)) than the medium process model. ME 2 was significant (*p* = 0.001) and Sample Both had a lower score across all levels of complexity than Sample Expert.

Regarding duration, ME 1 was significant and repeated contrasts showed that the medium process model (*p* = 0.001; $\eta_{p}^{2}=0.43$) had a longer duration (M = 47,008.29 (15,766.13)) than the easy process model (M = 31,549.98 (7761.69)) and the hard process model (*p* = 0.001; $\eta_{p}^{2}=0.28$) had a longer duration (M = 61,531.92 (17,697.44)) than the medium process model.

#### 3.1.3. Results for RQ 3

Table 5 shows the results for all performance measures (i.e., fixation, scan path, score, and duration) with respect to RQ 3.

Regarding fixation, ME 1 was significant and repeated contrasts showed that the medium process model (*p* = 0.001; $\eta_{p}^{2}=0.97$) had more fixations (M = 165.42 (10.16)) than the easy process model (M = 106.12 (5.67)) and the hard process model (*p* = 0.001; $\eta_{p}^{2}=0.94$) had more fixations (M = 216.16 (8.90)) than the medium process model.

Regarding fixation duration, ME 1 was significant and repeated contrasts showed that the medium process model (*p* = 0.001; $\eta_{p}^{2}=0.85$) had a longer average fixation (M = 216.57 (39.38)) duration than the easy process model (M = 205.13 (38.67) and the hard process model (*p* = 0.001; $\eta_{p}^{2}=0.32$) had a longer average fixation duration (M = 225.92 (38.67)) than the medium process model.

Regarding scan path, ME 1 was significant and repeated contrasts showed that the medium process model (*p* = 0.001; $\eta_{p}^{2}=0.95$) had longer scan paths (M = 38,954.61 (3004.47)) than the easy process model (M = 21,782.84), but the hard process model (*p* = 0.789; $\eta_{p}^{2}=0.00$) did not have longer scan paths (M = 39,133.30) than the medium process model.

Regarding score, neither ME nor IE reached significance.

Regarding duration, ME 1 was significant and repeated contrasts showed that the medium process model (*p* = 0.001; $\eta_{p}^{2}=0.49$) had a longer duration (M = 47,386.67 (15,208.31)) than the easy process model (M = 31,476.28 (8055.55)) and the hard process model (*p* = 0.001; $\eta_{p}^{2}=0.23$) had a longer duration (M = 61,039.81 (16,385.06)) than the medium process model.

## 4. Discussion

In RQ 1, we evaluated whether novices supported by EMMEs show better performance measures than novices not supported by EMMEs and whether this depends on the level of complexity of the process models. We found that novices supported by EMMEs had significantly better performance measures than novices not supported by EMMEs (i.e., *significant ME 2*). For most performance measures, this did not depend on the complexity of the process models (i.e., *non-significant IE*), except for the performance measures average fixation duration and score. Follow-up analyses showed that the interaction effect for both performance measures (i.e., average fixation duration and score) can be interpreted as follows: A more significant decrease in the performance measures is observable in novices not supported by EMMEs than in novices supported by EMMEs between the process models showing a medium and hard level of complexity. This indicates that EMMEs may prevent a decrease in these performance measures when it comes to hard process models. This might be explained that the support of EMMEs in process model comprehension leads to a reduction of the mental load, which is beneficial especially for complex process models with a high number of modeling elements and structures (e.g., loops) [42]. The shorter fixation durations in the group of novices supported by EMMEs were an additional indication that EMMEs support a reduction in the mental load [43]. This might be due to the fact that the application of EMMEs mainly target attention during process model comprehension to only relevant information. The working memory does not have to process and interpret irrelevant information allowing for a more efficient comprehension of process models. The attention through the eye movement is targeted by the EMMEs on relevant information, which also reduces processioning time of perceived information allowing for a more effective comprehension of process models. Based on the obtained results, the application of EMMEs in the context of process model comprehension fosters the comprehension of respective models significantly. The placed visual cues in the dot display condition and the visual guidance in the path display condition contribute positively to the comprehension of such models. Similar work conducted by the authors in [20] confirm the observation that visual cues are beneficial in process model comprehension. For future work, it would be therefore interesting to evaluate the association between the application of EMMEs and the effects on the working memory. Obtained eye tracking parameters (e.g., fixations) might serve as an indicator for different mental load during process model comprehension [44]. A follow-up study should evaluate whether the application EMMEs (i.e., visual guidance) also show a long-term effect. Do novices benefit in process model comprehension when the visual guidance (i.e., EMMEs) is removed from the process models?

In RQ 2, it was investigated whether novices supported by EMMEs show different performance measures as experts. Except for the performance measure score, novices supported by EMMEs and experts did not differ from each other (i.e., *non-significant ME 2*). With regard to scores, novices supported by EMMEs were worse than experts. A reason therefore might be that for the comprehension questions the process models must be memorized. As a consequence, there is an increasing risk that the answers were guessed due to inaccurate memorization [45]. Another explanation in this context is that experts are more effective in processing the presented information during process model comprehension due to their prior modeling experience. Research showed that novices and experts differ regarding problem solving and decision making [46]. It was shown that the experts’ knowledge is better structured in the individual and collective memory through deliberate practice, resulting in a more efficient access of respective knowledge in this context. The level of complexity of the process models did not influence this result as the IE did not reach significance. Further, in Study One, the following three performance measures between novices, which were not supported by EMMEs (i.e., Sample Novice), and experts (i.e., Sample Expert), showed significance: fixation (*p* = 0.008), score (*p* = 0.001), and duration (*p* = 0.013). The results indicated that experts performed better in the comprehension of process models [23]. However, in the current study, the respective performance measures showed no significance between novices, which were supported by EMMEs (i.e., Sample Both), and experts (i.e., Sample Expert). As a result, an EMME might enable a novice to achieve similar performance as experts in the comprehension of process models. Since performance measures regarding eye movements showed no significant differences, it could be that the novices’ attention was only focused on the visual cues (i.e., dots or path) in the process models. Potential relevant information from the process model was not considered, which would have been advantageous for novices regarding a proper process model comprehension (e.g., an activity before a decision), and were therefore recommended in order to answer a comprehension question. As a result and as shown in studies from other domains (e.g., [27,29], we want to emphasize the positive effects of the application of different types of EMMEs. However, there are other explanations as well. For example, the sample size of the novices in Study One (Sample Novice: N = 17) and in the current study (Sample Both: N = 43) were not the same and the sample size affects the probability to detect significant differences. Yet, as the sample size was larger in the current study, the probability would have been higher to detect differences with this larger sample compared to the smaller sample of novices recruited for Study One (see Section 4.2. Finally, it can also be evaluated in a future study whether experts in general may also benefit from the application of EMMEs in process models.

**Figure 5:**
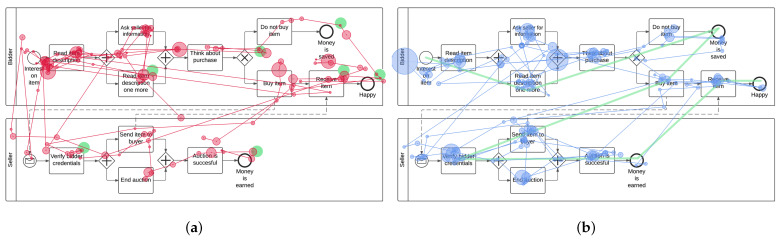
Scan paths for Eye Movement Modeling Examples. (**a**) Dot display condition; (**b**) Path display condition.

In RQ 3, we analyzed whether dot or path display conditions result in better performance measures. There were no significant differences between the two conditions (i.e., *non-significant ME 2*) and this result did not depend on the level of complexity of the process models (i.e., *non-significant IE*). The dot display condition mainly refers to process model syntactics (i.e., compliance with process modeling rules) with the provision of only visual cues in a process model, whereas the path display condition refers to process model semantics by denoting a given scan path in order to affect the reading direction. This is to ensure that all relevant information is considered during process model comprehension. However, the results confirm that both EMME conditions ensure that the syntactic as well as semantic dimension of a process model are properly captured and correctly comprehended by novices. A reason might be that both conditions raise awareness regarding the syntactic and semantic dimension in a process model. By directing the gaze of participants only to relevant information, more capacity remains free in the working memory that can be used to correctly interpret model semantics as well as syntactics. Finally, for illustration purposes, Figure 5 present recorded eye movements (i.e., scan path) of two participants, i.e., dot (see Figure 5a) and path display condition (see Figure 5b). Notably, similar and different eye movements can be distinguished in both conditions. In this context, the consideration of other EMME conditions (e.g., spotlight display condition, in which relevant parts in a stimulus are brighter and more visible while the other parts are darkened) may be subject of future work.

Generally, in all three RQs, the ME 1 attained significance in all performance measures except one (see Score in RQ 3) indicating that process models were more difficult to comprehend when they were more complex. The selected performance measures (see Section 2.4) in this study may be used as appropriate indicators evaluating, for example, confronted mental load during the comprehension of process models with varying complexity. Similar research also demonstrated (e.g., [14,47] that with rising level of complexity process model comprehension becomes more difficult. Rationales are, for example, the increasing number of modeling elements that needed to be comprehended or the more ramified structure (e.g., varying process flow direction) in larger process models.

### 4.1. Implications

The provided insights have implications for practice by demonstrating the applicability of EMMEs as well as for research on process model comprehension.

**For practice:** Since process models in real world are usually not provided with any visual guidance (i.e., EMME), the results have predominantly an impact on the formal training in the comprehension of such models. Process models may be enriched with visual cues guiding practitioners appropriately throughout a process model in order to ensure a proper comprehension. Depending on the focus set, the visual cues can be provided in such way that process model semantics (i.e., path display condition) or syntactics (i.e., dot display condition) are correctly captured and comprehended. Formal training in process model comprehension can benefit from the advantages of EMMEs to offer a more effective training [29]. Novices (e.g., doctors) can benefit from the application of EMMEs in order to develop a better understanding of working with process models [24]. Moreover, by capturing the attention with visual cues, crucial parts in a process model can be emphasized to highlight their importance. In this way, relevant information (i.e., about the who, where, and when) in a process can be extracted more efficiently without drawbacks regarding the mental load. Since EMMEs lead to a reduced load on the working memory (i.e., shorter average fixation duration) [48], practitioners can use the capacity in their working memory freed up by EMMEs for other tasks (e.g., more effective learning) [49]. Process modeling tools can be developed more specifically or can be extended with additional features to attract and guide the attention of practitioners on important elements or modeling constructs in a process model. The different EMME conditions (e.g., path or dot display condition) may be displayed permanently and according to the needs in order to ensure an optimized assistance during process model comprehension. In addition, the level of complexity of process models should be reduced in order to ensure a proper comprehension of such models. Approaches like changes in the visual representation (i.e., syntax modification) of a process model or the modularization of specific parts in a process model should be taken into account [50,51]. Finally, although the use of EMMEs was investigated with BPMN process models, they can be applied to other notations for process modeling (e.g., Event-driven Process Chains (EPCs)) as well.

**For research:** The insights from this study confirm the results from relevant other works on how to improve process model comprehension with visual cues [20], or by emphasizing the use of colors in the secondary notation [52]. Another question for research based on the obtained results is: does another type of EMME condition (e.g., spotlight display condition [28]) may have a different effect on process model comprehension? Further, the human brain operates in such way that highlighted visual information is perceived more dominantly compared to non-highlighted information [53]. It can also lead to circumstances where the non-highlighted information is not perceived at all, although it may be of importance (e.g., availability heuristic) [54]. Towards cognitive load, it would be interesting to investigate the differences in the cognitive load during process model comprehension, when confronting participants with the application of EMMEs and without the application of any EMMEs. This will allow for potential effects (e.g., availability heuristic) and their implications on process model comprehension to be investigated in more detail. The research question whether domain experts (e.g., juxtaposing doctors and economists) perceive EMMEs and respective condition differently could unravel new insights. For example, a doctor might better cope with the complexity of a process model when using the path display condition. The same applies for demographic characteristics such as age and gender. The question arises whether experts in process modeling may work more effectively by using EMMEs. Moreover, further research using interactive EMMEs may provide a new kind of guidance for practitioners in the comprehension of process models [55]. For example, colored dots in a process model that disappear once they have been viewed for a defined time and which appear in different positions to draw the attention on further important modeling structures (e.g., decisions in a process) in the model. Finally, another focus should be put on the inherent level of complexity of process models. With increasing process model complexity, more semantic and syntactic information needs to be extracted from a process model and stored in the working memory. As capacities in the working memory are limited, not all information can be stored and may be therefore not reflected properly [56]. This issue should be addressed on how to ensure a proper comprehension of this kind of information from a process model. Finally, this study showed that the application of EMMEs results in a shorter average fixation duration. According the work presented in [39], the fixation duration correlates with the cognitive load and longer fixation durations indicate an increased strain on the working memory. Additional research should aim to reduce the fixation duration in order to relieve the working memory during process model comprehension. On the other hand, a reduction of the number of fixations can be investigated as it is an indication for a high confronted cognitive complexity [57]. The reduction in both, fixation and respective duration, should lead to more available capacity in the working memory, enabling a more effective process model comprehension.

### 4.2. Limiting Factors

There are several limiting factors in this study that needed to be discussed and addressed in future studies. First, the used process models might not be representative. Process models document often complex procedures from the real world. However, the process models used in the study are of rather simple nature. Large and complex process models pose different demands on mental workload compared to simple process models. Second, participants of the study constitute another limitation. We tried to have a balanced and heterogeneous sample size, but, most participants were recruited from the field of computer science. Participants from other fields (e.g., healthcare) may perceive the application of EMMEs differently. In this context, significant differences in the baseline variables were found (see Table 1). The baseline variables related to gender and age reflect significant differences between participants. Consequently, obtained significant results in the application of EMMEs could also be the result of differences in these base variables (e.g., participants with an age < 25 years benefit more from the application of EMMEs juxtaposed with an age > 24 years). Third, the categorization of participants in the group of novices and experts with questions about prior modeling experience might be too vague and an additional expertise test might be better for a more precise categorization. Fourth, the documented scenarios in the process models constitute an additional risk. Familiar process scenarios might have a positive influence on the comprehension of process models in comparison with unfamiliar process scenarios. Fifth, the missing possibility to have a glance at the process model, while answering the comprehension questions, represents another limiting factor. The process models as well as documented scenarios must be kept in mind and the risk to guess an answer is growing due to an incomplete or either wrong memorization. Sixth, the sizes of the samples also limit the statistical power and there might be additional significant differences between the samples, which we could not show in this study, but which might become apparent in larger samples. Seventh, the results of the comparisons between the dot and path display conditions (RQ 3) have a higher internal validity than the results for RQ 1 and RQ 2, since the allocation of the participants to the two conditions for RQ 3 was done by the researcher (i.e., round-robin approach), whereas the control condition for RQ 1 (i.e., novices without EMMEs) as well as the control condition for RQ 2 (i.e., experts) were historical controls from a previous study. Differences in these samples might be confounders and might have influenced the results. Eighth, the comparison of data obtained between this study and a previous study (i.e., historical Study One) reflect another limitation. In more detail, although the procedure between the two studies has been kept the same, circumstances of data collection might have changed which could have lead to different results. Ninth, the factor process model complexity is not counter-balanced. The process model were presented in increasing level of complexity. Hence, lower performance measures in the more complex process models might be caused due to exhaustion of the participants. Tenth, the robustness of the provided comprehension benefits by the application of the EMMEs was not evaluated extensively. This means that the participants should have at least comprehended a process model without any visual guidance in order to evaluate the long-term effects of the EMMEs (i.e., may comprehension strategies be successfully transferred).

## 5. Conclusions and Future Work

**Figure 6:**
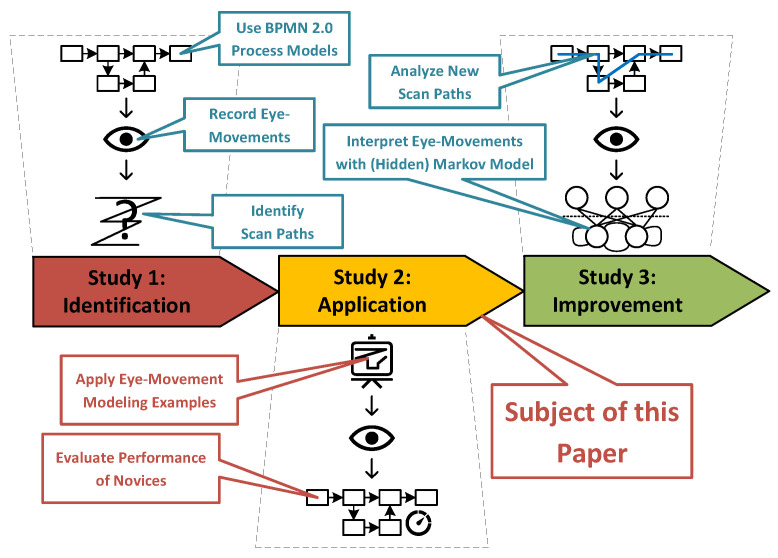
Three-stage study setting to foster process model comprehension.

This paper presented the insights obtained from a study in which observation capabilities of process modeling experts were conveyed to novices. In the scope of three research questions (i.e., RQ 1–RQ 3), Eye Movement Modeling Examples (EMMEs), reflecting two different conditions (i.e., dot and path display condition), were used to guide novices in the comprehension of process models. There were no significant differences regarding process model comprehension between both conditions. When juxtaposing condition results with results of novices and experts from the prior Study One, the results confirm that the application of EMMEs enhances process model comprehension significantly. Similar performance could even be achieved by novices compared to experts with the application of EMMEs in the context of process model comprehension. The results emphasize the application of EMMEs to foster process model comprehension in practice as well as research. For example, existing tools can be augmented with respective visual features for improving process model comprehension. Further, educational and formal training regarding the reading and comprehension of process models can be tailored more properly. We were able to highlight with this work the importance of EMMEs in the context of process model comprehension and to further study the role of the comprehension performance and the mental load in this context. This study is part of a three-stage study (see Figure 6) with the objective of providing directives as well as guidance enabling the better comprehension of process models. In a third study, recorded eye movements will be analyzed and interpreted using a (hidden) Markov model (HMM). More specifically, this question will be addressed: are there non-observable states (e.g., latent or subconscious) of eye movements (e.g., saccadic jumps) or strategies (e.g., holistic or analytic) that a HMM can successfully predict? Recent research demonstrated that HMM-based approaches may unravel new insights about cognitive functions (e.g., learning, decision making) [58,59]. A HMM allows for the identification of not yet considered commonalities as well as differences between novices and experts during the comprehension of process models, thereby enabling a better support in model comprehension [60]. All potential findings collected during the three studies can be used to foster process model comprehension, thereby having direct impacts on research and practice (see Figure 6). Our existing conceptual framework for the comprehension of process models that already incorporates methods and theories from cognitive neuroscience and psychology is therefore enriched by the findings of this work [45].

## Figures and Tables

**Table 1:** Sample description and comparison in baseline variables.

Variable	Dot (N=21)	Path (N=22)	Novice (N=17)	Expert (N=19)	Significance
Gender N (%)					
female	9 (42.9)	9 (40.9)	7 (41.2)	2 (10.5)	
male	12 (57.1)	13 (59.1)	10 (58.8)	17 (89.5)	p=0.003 ${}^{a}$
Age (year), mean (SD)	23.05 (1.94)	22.36 (2.25)	30.8 (7.2)	26.3 (4.1)	
Age N (%)					
<25 years	16 (76.2)	18 (81.8)	3 (17.6)	6 (31.6)	
>24 years	5 (23.8)	4 (18.2)	14 (82.4)	13 (68.4)	p=0.001 ${}^{a}$
Experience N (%) ^+^					
0 (No at all)	14 (66.7)	9 (40.9)	5 (29.4)	0 (0)	
1 (Slightly)	7 (33.3)	13 (59.1)	12 (70.6)	0 (0)	
2 (Somewhat)	0 (0)	0 (0)	0 (0)	7 (36.8)	
3 (Moderately)	0 (0)	0 (0)	0 (0)	4 (21.1)	
4 (Highly)	0 (0)	0 (0)	0 (0)	8 (42.1)	p=0.001 ${}^{a}$

^+^ What is your experience in process modeling?; ${}^{a}$ Fisher’s exact test.

**Table 2:** Descriptive results for each sample.

	Descriptive Statistics for Sample Dot					
	**Easy**		**Medium**		**Hard**	
	**M**	**SD**	**M**	**SD**	**M**	**SD**
Fixation	105.33	(5.04)	166.48	(9.53)	218.05	(8.87)
Fixation Dur.	204.43	(39.61)	215.91	(41.34)	223.06	(36.92)
Scan Path	22,263.29	(1764.72)	38,999.76	(2805.71)	39,766.38	(3200.12)
Score	2.81	(0.68)	2.76	(0.77)	2.57	(0.87)
Duration	31,875.19	(8976.25)	47,941.10	(15,709.23)	58,472.90	(15,411.26)
	**Descriptive Statistics for Sample Path**					
	**M**	**SD**	**M**	**SD**	**M**	**SD**
Fixation	106.86	(6.24)	164.41	(10.83)	214.36	(8.76)
Fixation Dur.	205.80	(38.68)	217.21	(38.39)	228.65	(40.95)
Scan Path	21,324.23	(2039.13)	38,911.50	(3248.47)	38,529.00	(3393.92)
Score	3.14	(0.71)	3.09	(0.75)	2.77	(0.69)
Duration	31,095.50	(7261.99)	46,857.45	(16,065.06)	64,099.90	(19,927.83)
	**Descriptive Statistics for Sample Both**					
	**M**	**SD**	**M**	**SD**	**M**	**SD**
Fixation	106.12	(5.67)	165.42	(10.16)	216.16	(8.89)
Fixation Dur.	205.13	(38.67)	216.57	(39.38)	225.92	(38.67)
Scan Path	21,782.84	(1946.17)	38,954.61	(3004.47)	39,133.30	(3320.78)
Score	2.98	(0.71)	2.93	(0.76)	2.67	(0.78)
Duration	31,476.28	(8055.55)	47,386.67	(15208.31)	61,039.81	(16,385.06)
	**Descriptive Statistics for Sample Novice**					
	**M**	**SD**	M	**SD**	**M**	**SD**
Fixation	138.71	(41.10)	209.00	(56.37)	264.53	(96.51)
Fixation Dur.	222.51	(53.65)	239.20	(45.80)	258.58	(60.65)
Scan Path	26,989.24	(6200.60)	47,298.94	(18,666.35)	49,834.94	(37,663.70)
Score	2.09	(1.02)	1.98	(1.04)	0.92	(0.52)
Duration	38,549.76	(14,226.58)	63,928.06	(23,057.75)	73,465.47	(24,962.57)
	**Descriptive Statistics for Sample Expert**					
	**M**	**SD**	M	**SD**	**M**	**SD**
Fixation	104.05	(34.34)	167.26	(47.80)	206.00	(56.04)
Fixation Dur.	198.99	(37.19)	214.12	(39.12)	218.80	(42.92)
Scan Path	22,954.89	(11,107.35)	37,043.32	(17,865.93)	40,058.21	(13,787.34)
Score	3.74	(0.65)	3.47	(0.70)	2.95	(0.78)
Duration	31,716.79	(7259.66)	46,151.95	(17,366.06)	62,645.63	(20,810.57)

**Table 3:** Results of inferential statistics for RQ 1.

	Fixation				Fixation Duration			
ME 1	F(1.78; 103.41)	=232.40;	*p* = 0.001;	$\eta_{p}^{2}=0.80$	F(1.35; 78.34)	=61.10;	*p* = 0.001;	$\eta_{p}^{2}=0.51$
ME 2	F(1; 58)	=24.18;	*p* = 0.001;	$\eta_{p}^{2}=0.29$	F(1; 58)	=4.00;	*p* = 0.050;	$\eta_{p}^{2}=0.07$
IE	F(1.78; 103.41)	=1.09;	*p* = 0.334;	$\eta_{p}^{2}=0.18$	F(1.51; 78.34)	=4.55;	*p* = 0.025;	$\eta_{p}^{2}=0.07$
	**Scan Path**				**Score**			
ME 1	F(1.28; 74.12)	=36.31;	*p* = 0.001;	$\eta_{p}^{2}=0.39$	F(1.92; 111.58)	=15.11	*p* = 0.001;	$\eta_{p}^{2}=0.21$
ME 2	F(1; 58)	=13.52;	*p* = 0.001;	$\eta_{p}^{2}=0.19$	F(1; 58)	=63.14;	*p* = 0.001;	$\eta_{p}^{2}=0.52$
IE	F(1.28; 74.12)	=0.55;	*p* = 0.504;	$\eta_{p}^{2}=0.01$	F(1.92; 111.58)	=5.42;	*p* = 0.006;	$\eta_{p}^{2}=0.09$
	**Duration**				ME 1 = Main effect complexity;			
ME 1	F(1.65; 95.86)	=52.01;	*p* = 0.001;	$\eta_{p}^{2}=0.47$	ME 2 = Main effect sample comp.;			
ME 2	F(1; 58)	=18.61;	*p* = 0.001;	$\eta_{p}^{2}=0.24$	IE = Interaction effect compl.*samp.			
IE	F(1.65; 95.86)	=1.01;	*p* = 0.328;	$\eta_{p}^{2}=0.02$				

**Table 4:** Results of inferential statistics for RQ 2.

	Fixation				Fixation Duration			
ME 1	F(1.79; 107.25)	=430.36;	*p* = 0.001;	$\eta_{p}^{2}=0.88$	F(1.46; 87.47)	=56.55;	*p* = 0.001;	$\eta_{p}^{2}=0.49$
ME 2	F(1; 60)	=0.33;	*p* = 0.568;	$\eta_{p}^{2}=0.01$	F(1; 60)	=0.25;	*p* = 0.622;	$\eta_{p}^{2}=0.01$
IE	F(1.79; 107.25)	=1.43;	*p* = 0.245;	$\eta_{p}^{2}=0.02$	F(1.46; 87.47)	=0.81;	*p* = 0.415;	$\eta_{p}^{2}=0.01$
	**Scan Path**				**Score**			
ME 1	F(1.61; 96.81)	=167.98;	*p* = 0.001;	$\eta_{p}^{2}=0.74$	F(2; 119.98)	=8.60;	*p* = 0.001;	$\eta_{p}^{2}=0.13$
ME 2	F(1; 60)	=0.00;	*p* = 0.975;	$\eta_{p}^{2}=0.00$	F(1; 60)	=16.24;	*p* = 0.001;	$\eta_{p}^{2}=0.21$
IE	F(1.61; 96.81)	=1.36;	*p* = 0.259;	$\eta_{p}^{2}=0.02$	F(2; 119.98)	=1.62;	*p* = 0.203;	$\eta_{p}^{2}=0.03$
	**Duration**				ME 1 = Main effect complexity;			
ME 1	F(1.64; 98.60)	=60.51;	*p* = 0.001;	$\eta_{p}^{2}=0.50$	ME 2 = Main effect sample comp.;			
ME 2	F(1; 60)	=0.01;	*p* = 0.933;	$\eta_{p}^{2}=0.00$	IE = Interaction effect compl.*samp.			
IE	F(1.64; 98.60)	=0.13;	*p* = 0.835;	$\eta_{p}^{2}=0.00$				

**Table 5:** Results of inferential statistics for RQ 3.

	Fixation				Fixation Duration			
ME 1	F(1.79; 73.26)	=2077.69;	*p* = 0.001;	$\eta_{p}^{2}=0.98$	F(1.18; 48.37)	=74.17;	*p* = 0.001;	$\eta_{p}^{2}=0.64$
ME 2	F(1; 41)	=0.72;	*p* = 0.401;	$\eta_{p}^{2}=0.03$	F(1; 41)	=0.05;	*p* = 0.817;	$\eta_{p}^{2}=0.00$
IE	F(1.79; 73.26)	=1.22;	*p* = 0.298;	$\eta_{p}^{2}=0.02$	F(1.18; 48.37)	=1.03;	*p* = 0.326;	$\eta_{p}^{2}=0.03$
	**Scan Path**				**Score**			
ME 1	F(1.80; 73.63)	=513.31;	*p* = 0.001;	$\eta_{p}^{2}=0.93$	F(1.98; 81.15)	=2.26;	*p* = 0.112;	$\eta_{p}^{2}=0.05$
ME 2	F(1; 41)	=2.56;	*p* = 0.117;	$\eta_{p}^{2}=0.06$	F(1; 41)	=3.92;	*p* = 0.054;	$\eta_{p}^{2}=0.09$
IE	F(1.80; 73.63)	=0.46;	*p* = 0.613;	$\eta_{p}^{2}=0.01$	F(1.98; 81.15)	=0.12;	*p* = 0.890;	$\eta_{p}^{2}=0.00$
	**Duration**				ME 1 = Main effect complexity;			
ME 1	F(1.58; 64.74)	=44.07;	*p* = 0.001;	$\eta_{p}^{2}=0.52$	ME 2 = Main effect sample comp.;			
ME 2	F(1; 41)	=0.24;	*p* = 0.624;	$\eta_{p}^{2}=0.01$	IE = Interaction effect compl.*samp.			
IE	F(1.58; 64.74)	=0.60;	*p* = 0.515;	$\eta_{p}^{2}=0.01$				

## Data Availability

The data presented in this study are available on request from the corresponding author.

## Abbreviations

The following abbreviations are used in this manuscript:

BPMN	Business Process Model and Notation
EMME	Eye Movement Modeling Example
HMM	(Hidden) Markov Model
IE	Interaction Effect
KPI	Key Performance Indicator
ME	Main Effect
RQ	Research Question

## Appendix A

Study materials (i.e., EMMEs and comprehension questions) can be found at:

https://drive.google.com/drive/folders/14kLZTIujjbrtpvJqof7urLa2m4gF9up_?usp=sharing
